# Physical Treatments Modified the Functionality of Carrot Pomace

**DOI:** 10.3390/foods13132084

**Published:** 2024-07-01

**Authors:** Jordan Richards, Amy Lammert, Jack Madden, Iksoon Kang, Samir Amin

**Affiliations:** 1 Food Science and Nutrition Department, California Polytechnic State University, San Luis Obispo, CA 93407, USA; jricha@calpoly.edu (J.R.); alammert@calpoly.edu (A.L.);; 2 Animal Science Department, California Polytechnic State University, San Luis Obispo, CA 93407, USA; ikang01@calpoly.edu

**Keywords:** carrot pomace, food waste, functional properties, freeze-drying, dehydration, dietary fibers, carotenoids

## Abstract

This study addressed the critical issue of food waste, particularly focusing on carrot pomace, a by-product of carrot juice production, and its potential reutilization. Carrot pomace, characterized by high dietary fiber content, presents a sustainable opportunity to enhance the functional properties of food products. The effects of physical pretreatments—high shearing (HS), hydraulic pressing (HP), and their combination (HSHP)—alongside two drying methods (freeze-drying and dehydration) on the functional, chemical, and physical properties of carrot pomace were explored. The results indicated significant enhancements in water-holding capacity, fat-binding capacity, and swelling capacity, particularly with freeze-drying. Freeze-dried pomace retained up to 33% more carotenoids and demonstrated an increase of up to 22% in water-holding capacity compared to dehydrated samples. Freeze-dried pomace demonstrated an increase of up to 194% in fat-binding capacity compared to dehydrated samples. Furthermore, HSHP pretreatment notably increased the swelling capacity of both freeze-dried (54%) and dehydrated pomace (35%) compared to pomace without pretreatments. Freeze-drying can enhance the functional properties of dried carrot pomace and preserve more carotenoids. This presents an innovative way for vegetable juice processors to repurpose their processing by-products as functional food ingredients, which can help reduce food waste and improve the dietary fiber content and sustainability of food products.

## 1. Introduction

Approximately one-third of all food produced globally is lost or wasted somewhere along the food supply chain [1]. Food loss and waste have been reported to occur throughout the food-processing cycle; this includes everything from in-field harvest to processing and packaging facilities and retail grocery stores. This represents a waste of the water, land, energy, and natural resources used to produce food and is estimated to cause USD 940 billion in economic losses and produce more than 4.4 gigatons of greenhouse gas emissions (CO_2_ equivalent) annually [1]. The United States Environmental Protection Agency (EPA) estimates that annual food loss and waste are equivalent to 170 million metric tons of CO_2_ equivalent emissions within the U.S. [2]. Reducing food waste within the U.S. presents opportunities to address climate change, conserve resources, and increase food security, productivity, and economic efficiency. According to the United States Department of Agriculture (USDA), 31% of food is wasted, amounting to a total of USD 218 billion, or 1.3% of the country’s Gross Domestic Product (GDP).

In 2019, the total production of carrots in the U.S. reached 2.53 million metric tons, which was a 13% increase from the 2018 total [3]. Carrots are the sixth-most consumed fresh vegetable in the U.S. [3]. Per capita consumption of fresh carrots in the U.S. peaked at 6.4 kg in 1997 and then decreased to around 3.8 kg in 2022 [4]. Over the past 35 years, the U.S. carrot industry has changed with the introduction of fresh-cut technology for more value-added carrot products such as pre-cut carrots, baby carrots, and carrot juice, which has increased the amount of carrot waste from carrot processing. When producing carrot juice, a pulp by-product is generated that is equivalent to 50% of the raw material [5]. Carrot by-products are rich in bioactive substances such as carotenoids (especially β-carotene), insoluble and soluble fiber is composed of pectic polysaccharides, hemicellulose and cellulose [5,6].

Using carrot pomace reduces food waste and produces functional ingredients for the food industry [7]. Carrot pomace contains approximately 55% dietary fiber, which could increase water-holding capacity from 17.9 to 23.3 g water/g fiber [7,8]. Dietary fiber can also hold fat particles and play a key functional role in foods [9]. Fat-binding capacity (FBC) and WHC are important for improving product quality, such as juiciness, flavor, and mouthfeel [10].

Drying is the process of removing moisture from a material via natural or unnatural conditions. Drying technologies for fruits and vegetables include hot air drying, microwave drying, vacuum drying, freeze-drying, and heat pump drying [10]. Drying is a frequently used method to reduce volume and weight, therefore reducing the costs of packaging, storage, and transportation. Drying can also affect the flavor and textural properties of fruits and vegetables [11]. Dehydration is, by definition, the removal of water via evaporation from solid or liquid food to obtain a solid product with low water activity to inhibit microbial growth [12]. Drying methods influence food products’ density, porosity, and rehydration features. Convective drying can reduce hydrophilic properties due to irreversible cellular rupture, resulting in dense structure and integrity losses by broken and shrunken capillaries, which hinders water absorption and rehydration. Freeze-dried fruits and vegetables are usually characterized by minimal shrinkage and less structural collapse due to their highly porous structures after water removal via sublimation [13,14,15]. Different drying methods resulted in different porous structures, and freeze-drying produced higher porosity in food structures (80–90%) [13]. Microwave-dried potato and carrot had a porosity of approximately 75%, while vacuum-drying decreased the porosity to 50% in carrot and 25% in potato [13,16].

The objective of this study is to investigate how pretreatments such as high-shear mixing, hydraulic pressing, and a combination of both, followed by drying methods such as dehydration and freeze-drying, affect the functional properties of carrot pomace. Specifically, the study aims to evaluate the impact of these treatments on water-holding capacity, fat-binding capacity, swelling capacity, dietary fiber composition, and carotenoid content. This presents an innovative way for vegetable juice processors to repurpose their processing by-products as functional food ingredients, which can help reduce food waste and improve the dietary fiber content and sustainability of food products.

## 2. Materials and Methods

Carrot pomace was obtained from Grimmway Family Farms (Arvin, CA, USA). Carrot pomace was placed in 22 kg sealed, food-grade pails and stored in a dark freezer at −20 °C until further processed. Freezing pomace prior to processing minimized chances of microbial growth and degradation of carotenoids.

### 2.1. Mechanical Pretreatments of Carrot Pomace

The frozen carrot pomace was thawed overnight in a refrigerated room and pretreated using one of the three methods prior to drying: (1) high-shear (HS) for 5 min @ 15,000 RPM (Yuchengtech AD300L-H High-Shear Mixer, Shanghai, China), (2) hydraulic press (HP) (Hydraulic Wells Juice Press, Samson Brands, Danbury, CT, USA), and (3) the combination of high-shear and hydraulic press (HSHP).

### 2.2. Mechanical Drying Treatment of Carrot Pomace

Carrot pomace with and without pretreatment was dried using one of two methods: (1) dehydration (D) using a drying oven (Harvest Saver R4 drying oven, Commercial Dehydrator Systems, Inc., Eugene, OR, USA) at 40 °C for 24 h on fan speed 1 (0.13 m/s) and (2) lyophilization, or free drying (FD), using a freeze-dryer (Harvest Right Freeze Dryer, Salt Lake City, UT, USA) at −20 °C and 6.67 Pa for 24 h (Table 1). Non-pretreated and pretreated dried carrot pomace was then ground using a commercial spice grinder (VEVOR 2500 g Electric Grain Mill Grinder, Sacramento, CA, USA) to pass through a 20-mesh sieve (0.85 mm) and stored at −22 °C after placing into gallon-sized plastic bags (Ziplock, SC Johnson & Sons, Inc., Racine, WI, USA) wrapped in aluminum foil (Reynolds Wrap Reynolds, Consumer Products, Lake Forest, IL, USA).

### 2.3. Chemical Properties

#### 2.3.1. Total Moisture

Total moisture content was determined for both solid and liquid fractions of the carrot pomace. Approximately 2.50 g of carrot pomace was weighed, recorded, and placed in the Ohaus MB45 Moisture Analyzer (Ohaus Corp., Parsippany, NJ, USA) at 105 °C until no weight change was detected. The moisture content was determined using the following equation:

$$\mathrm{Moisture}\;\mathrm{Content}\;(\%)=\frac{\mathrm{Dried}\;\mathrm{weight}\;\mathrm{of}\;\mathrm{sample}\;(g)}{\mathrm{Weight}\;\mathrm{of}\;\mathrm{initial}\;\mathrm{sample}\;(g)}\times 100$$

#### 2.3.2. Carotenoid Content

Carotenoid contents were determined for carrot pomace samples (Table 1) according to the method described by Amin [17]. One gram of each carrot pomace sample (Table 1) was added to 25 mL of extraction solvent and homogenized for 30 s at 7500 rpm (Senstry Cyclone I.Q. 2 Sentry Microprocessor Digital Homogenizer, SP Industries Inc., Warminster, PA, USA) in 50 mL centrifuge tubes. The centrifuge tubes were centrifuged for 5 min at 6500 rpm and 5 °C (Eppendorf 5810 R Centrifuge, Hauppauge, NY, USA). After centrifuging, the supernatant layer containing hexane and non-polar carotenoids (β-carotene) was transferred to a 25.00 mL volumetric flask. The supernatant volume was adjusted to 25.00 mL with additional hexane. Absorbance values were measured at λmax450 nm (Shimadzu UV–1900 UV-VIS spectrophotometer, Shimadzu, MD, USA). An extinction coefficient of 2505 for β-carotene was used to calculate the concentration of carotenoids in the samples using Beer’s law.

#### 2.3.3. Total Dietary Fiber

**Figure 1:**
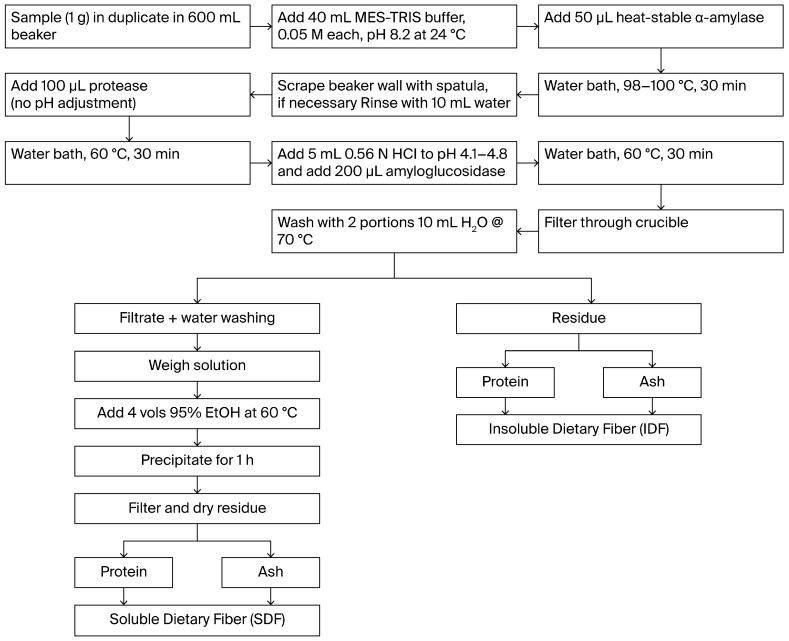
Total dietary fiber process flowchart from Megazyme [20].

Total dietary fiber (TDF), soluble dietary fiber (SDF), and insoluble dietary fiber (IDF) were determined for all pretreated and dried carrot pomace samples (Control, HSD, HSFD, HPD, HPFD, HSHPD, and HSHPFD) using the Megazyme total dietary fiber assay kit (K-TDFR-200A, Neogen, Lansing, MI, USA; Megazyme, Wicklow, Ireland) with modifications of AOAC 991.43 [18] and AACC 32–07.01 [19] (Figure 1). Samples were incubated with 50 mL of heat-stable alpha-amylase (Megazyme cat. no. E-BLAAM) (100 °C, 30 min) and then enzymatically digested with 100 mL protease (Megazyme cat. No. E-BSPRT) (60 °C, 30 min), followed by incubation with 200 mL of amyloglucosidase (Megazyme cat. No. E-AMGDF) (60 °C, 30 min) to remove protein and starch. The samples were filtered, washed (with water, 95% ethanol, and acetone), dried, and weighed to determine insoluble fiber (IDF). Four volumes of 95% ethanol (preheated to 60 °C) were added to the filtrate and the wash water. The precipitates were filtered and washed with 78% ethanol. The residues of soluble dietary fiber (SDF) were dried and weighed. The obtained values were corrected for ash and protein. TDF was determined by summing insoluble IDF and SDF. Fiber ratios were calculated as a ratio of IDF:SDF. Total dietary fiber was calculated using the equation below.

$$\mathrm{Dietary}\;\mathrm{Fiber}\;(\%)=\frac{\frac{R_{1}+R_{2}}{2}-P-A-B}{m_{1}}\times 100$$

R_1_ = IDF residue weight.

R_2_ = SDF residue weight.

m_1_ = sample weight.

A = ash weight from R_1_.

P = protein weight from R_2_.

B = blank.

#### 2.3.4. Amylase Neutral Detergent Fiber

Amylase neutral detergent fiber (aNDF) was determined for control dehydrated (CD) and freeze-dried (CFD) carrot pomace samples. The amounts of 0.45–0.55 g of sample and 0.5 g of sodium sulfite (Na_2_SO_3_) were weighed and combined. The samples were heated until boiling in 50 mL of neutral detergent solution. An amount of 2 mL of α-amylase was added before the beaker was heated. The sample was boiled for 1 h and filtered using a pretared fritted glass crucible. Fritted crucibles containing aNDF residue were dried at 100 °C for 24 h. The residue weight was then recorded. All samples were analyzed in triplicate.

#### 2.3.5. Acid Detergent Fiber

For sequential analysis of acid detergent fiber (ADF), the crucible containing the aNDF fiber preparation was analyzed sequentially. The crucible was placed on its side in a 600 mL Berzelius beaker, and the sample was boiled in 200 mL of acid detergent solution for 1 h. At the end of boiling, the crucible was removed with tongs, and the solution was gravimetrically transferred and filtered through the fritted crucible. Fritted crucibles containing ADF residue were dried at 100 °C for 24 h. The residue weight was then recorded. All samples were analyzed in triplicate.

### 2.4. Functional Properties

Functional properties were evaluated for all carrot pomace samples after drying using two methods (Table 1).

#### 2.4.1. Water-Holding Capacity

Water-holding capacity (WHC) was determined according to the method described by Raghavendra et al. [21]. Dried carrot pomace (0.50 g) was added to 15.00 mL of water in a graduated cylinder and mixed. After storing at ambient temperature for 24 h, the supernatant was filtered through a sintered glass crucible under vacuum. The hydrated residue weight was recorded before being dried at 105 °C for 1 h to obtain the residue dry weight. The water-holding capacity was measured as one gram of water held by one gram of pomace and calculated using the equation below.

$$\mathrm{WHC}\;(\frac{g\;\mathrm{water}}{g\;\mathrm{dry}\;\mathrm{pomace}})=\frac{\left(\mathrm{residue}\;\mathrm{hydrated}\;\mathrm{weight}-\mathrm{residue}\;\mathrm{dry}\;\mathrm{weight}\right)}{\left(\mathrm{residue}\;\mathrm{dry}\;\mathrm{weight}\right)}$$

#### 2.4.2. Fat-Binding Capacity

Fat-binding capacity (FBC) was determined according to Beuchat’s method [22] with modification. Canola oil (5.60 g) was added to dehydrated dried carrot pomace (1.00 g) in a 50 mL centrifuge tube. Due to the increased volume of freeze-dried pomace, the weight of the pomace used was reduced from 1.00 g to 0.10 g. Canola oil (5.60 g) was added to freeze-dried pomace in a 50 mL centrifuge tube. Each slurry was vortexed for 30 s, allowed to sit for 30 min at 22 °C, and then centrifuged at 1610× *g* for 25 min. The supernatant was decanted from the sample, the weight of the decanted sample was determined, and grams of oil retained per gram of sample was calculated. The fat-binding capacity was calculated using the equation below.

$$\mathrm{Fat}\;\mathrm{Binding}\;\mathrm{Capacity}\;\left(\frac{g}{g}\right)=\frac{\mathrm{Weight}\;\mathrm{of}\;\mathrm{decanted}\;\mathrm{sample}}{\mathrm{Weight}\;\mathrm{of}\;\mathrm{initial}\;\mathrm{sample}}$$

#### 2.4.3. Swelling Capacity

Swelling capacity was determined according to the method of Raghavendra et al. [21]. A total of 25 mL of deionized water was added to 1.00 g of dried carrot pomace in a 50.00 mL graduated cylinder. Graduated cylinders were covered with parafilm to reduce evaporation, and the samples were allowed to sit at 22 °C for 24 h. After 24 h, the volume of the swollen sample was measured. The swelling capacity was expressed as mL of water per 1.00 g of carrot pomace and was calculated using the equation below.

$$\mathrm{Swelling}\;\mathrm{Capacity}\left(\frac{\mathrm{mL}}{g}\right)=\frac{\mathrm{Volume}\;\mathrm{occupied}\;\mathrm{by}\;\mathrm{sample}}{\mathrm{Original}\;\mathrm{sample}\;\mathrm{weight}}$$

### 2.5. Statistical Analysis

Results of chemical and physical properties are reported as mean ± standard deviation. Two-way analysis of variance (ANOVA) was used to determine significant differences between functional properties based on the drying method and pretreatment using JMP Pro version 17 statistics software (Cary, NC, USA). Tukey’s post hoc analysis was performed to identify significant differences between treatments at *p* ≤ 0.05.

## 3. Results and Discussion

### 3.1. Characterization of Carrot Pomace

#### 3.1.1. Total Moisture and Solids Content

Hydraulic pressing (HP) and high-shearing/hydraulic pressing pretreatments (HSHP) significantly (*p* ≤ 0.05) reduced the moisture content of carrot pomace compared to the control and high-shear (HS) pretreatment (Table 2). HP increased the total solids content by 157% compared to the control.

The moisture content of whole carrots has been reported to be in the range of 86–89% [23], while the moisture content of carrot pomace has been reported as approximately 85% [24]. The application of hydraulic and expeller pressing significantly decreased the moisture content of commercially produced carrot mash by 9.10% and 12.56%, respectively [17]. The application of HP decreased carrot pomace’s moisture content by 9.29%, while HS had no significant impact, while the combination of HSHP pomace only decreased the moisture content by 5.03%. The difference observed can be attributed to the expeller press being able to apply high shear and compression simultaneously.

HSHP pretreatment could increase the soluble fiber content of carrot pomace. Soluble fibers have demonstrated the capacity to improve viscosity, gel-formation, and emulsification [25]. This could be why HSHP had a 4% higher moisture content than HP. The physical action of water removal could reduce drying time and increase the solids content of dried carrot pomace during processing.

#### 3.1.2. Total Carotenoid Content

The drying method significantly impacted the total carotenoid concentration of dried carrot pomace (*p* < 0.05). Freeze-drying significantly increased the total carotenoid concentration of carrot pomace compared to dehydration (Table 3).

Carotenoids are sensitive to quality loss by heat, light, and oxygen. During drying, the isomerization and oxidation of carotenoids can cause thermal degradation and quality degradation in color, flavor, and nutritional quality [26]. Carrot pomace that has not undergone pretreatments and drying contains 144.64 μg/g of carotenoids [27]. Both drying methods decreased carotenoids in dried pomace. Freeze-drying resulted in a 30.8%–53.1% reduction in carotenoids compared to untreated and undried pomace. Dehydration resulted in a 66.3%–75.5% reduction in carotenoids compared to untreated and undried pomace. Freeze-drying can increase the stability of carotenoids by reducing heat exposure and the oxidation rate at low temperatures and pressure [28]. Air-dried purple carrots had a 36.2% decrease in carotenoid content compared to freeze-dried purple carrots, which had a small decrease [29]. Freeze-dried carrot pomace subjected to high-shearing (HS and HSHP) showed a 33% reduction in carotenoid concentration compared to the control (*p* ≤ 0.05). Disruptions in cell walls due to shearing can enhance the release of phytochemicals such as carotenoids from the solid matrix. These phytochemicals could be released into the liquid fraction of the material. The bioavailability of carotenoids has been shown to increase with heating or cell wall disruption through chopping or shearing. This is consistent with the previous reports that attributed higher values to the high-shear’s ability to break down and release trapped carotenoid crystals within the cells [17,28,29,30].

#### 3.1.3. Fiber Composition

Drying methods and pretreatment application significantly modified the total dietary fiber (TDF), neutral detergent fiber (aNDF), and acid detergent fiber (ADF) contents (*p* ≤ 0.05) of carrot pomace (Table 4 and Table 5). Both the pretreatment and drying methods used had a significant effect on the levels of TDF (*p* ≤ 0.0001 and 0.0005, respectively). The interaction between the pretreatment and drying method was also significant (*p* ≤ 0.01) and impacted TDF levels. Similarly, both the pretreatment and drying methods had a statistically significant effect on IDF (insoluble dietary fiber) levels (*p* ≤ 0.0001). The interaction between the pretreatment and drying methods also significantly influenced IDF levels (*p* ≤ 0.0001). Furthermore, the pretreatment had a statistically significant effect (*p* = 0.0049) on SDF (soluble dietary fiber) levels. Additionally, the drying method had a significant impact on SDF levels (*p* ≤ 0.0001). Finally, the interaction between the pretreatment and drying methods significantly impacted SDF levels (*p* < 0.0058).

Physical pretreatments significantly affected the ratio of insoluble and soluble dietary fiber. Overall, freeze-dried samples showed a decrease in IDF and an increase in SDF in all pretreatments. The higher soluble fiber after physical shearing or pressing is presumed from the chemical interactions between insoluble fractions, hemicellulose, and lignin, which could convert insoluble fibers to soluble fibers [31].

Pretreatments had a more significant impact on TDF (total dietary fiber) compared to the drying methods and the combination of drying methods and pretreatments. The drying method alone had a more significant effect on SDF (soluble dietary fiber) compared to the pretreatment and the combination. On the other hand, the interaction between the drying method and pretreatment had a more significant effect on IDF (insoluble dietary fiber) than either factor alone.

Freeze-drying increased the aNDF and ADF contents compared to dehydration (Table 5). Freeze-dried carrot pomace showed a 32% increase in aNDF and a 43% increase in ADF compared to dehydrated pomace. Amylase neutral detergent fiber accounts for the hemicellulose, cellulose, and lignin present in the product, while ADF accounts for the removal of hemicellulose. In freeze-dried carrot pomace, cellulose and lignin made up 81% and 73% of the fiber content, respectively, compared to dehydrated pomace.

The total dietary fiber (TDF) content was higher in freeze-dried carrot pomaces (71.86 g/100 g) than in dehydrated carrot pomaces (51.84 g/100 g). These results indicated that the content of insoluble dietary fiber was higher in freeze-dried pomace (55.38 g/100 g), whereas the content of soluble fiber was higher in dehydrated pomace (20.70 g/100 g) than in freeze-dried pomace (16.49 g/100 g). Thermal processes have been an important factor in modifying insoluble and soluble fiber ratios and physiochemical properties [32]. The quantity of soluble fiber is generally influenced by the processing temperatures. Higher temperatures can break down glycosidic bonds in polysaccharides, lead to an increase in oligosaccharides, and, therefore, increase the quantity of soluble dietary fiber [33]. This may explain the increase of soluble fiber found in dehydrated pomaces. Various thermal treatments were shown to alter the insoluble and soluble ratio of barley fiber [34].

Pretreatments modified the TDF content in dehydrated and freeze-dried carrot pomaces (Table 6). HSHP pretreatment significantly increased the TDF content of dehydrated pomace from 52 g/100 g to 68.22 g/100 g. Dehydrated HS and HP pomace showed no significant difference in TDF compared to dehydrated control samples. However, HS and HP pretreatments decreased the TDF in freeze-dried carrot pomace. Pretreatments significantly affected the ratio of insoluble and soluble dietary fibers. Freeze-drying decreased the IDF content and increased the SDF content in all pretreatments. The higher soluble fiber in pomace after physical shearing or pressing is presumed from the chemical interactions that might convert the insoluble fractions to soluble fractions [31]. Physical treatments such as ball milling resulted in the redistribution of TDF in grape pomace and grape pomace fiber concentrate, causing an increase in SDF and a decrease in IDF [31].

The IDF/SDF ratio is an important factor as both fractions are complementary in their functional properties. As an acceptable food ingredient, the IDF/SDF ratio should be approximately 2:1 [35]. Carrot pomace dietary fiber could be a high-quality food ingredient due to the physiological effects of soluble and insoluble fibers. The total dietary fiber of carrot pomace has been reported to be 63.6%, with insoluble and soluble fractions of 50.1% and 13.5%, respectively [36].

### 3.2. Functional Properties of Carrot Pomace

#### 3.2.1. Water-Holding Capacity

**Figure 2:**
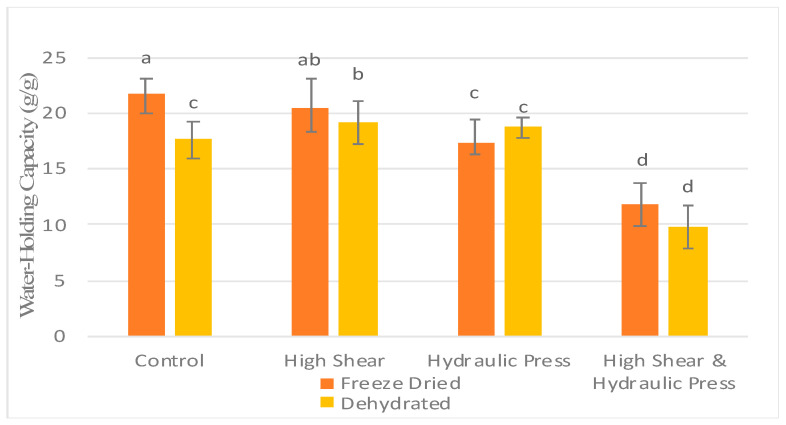
Impact of physical treatment methods on the water-holding capacity of commercially produced carrot pomace. ^a–d^ Different letters within the same physical treatment indicate significant differences at *p* ≤ 0.05.

When looking at the effect of the drying method, pretreatment or the interaction between the drying method and pretreatment (F = 43.215) had a more significant effect on WHC than the drying method (F = 1.474) and interactions between pretreatments and drying methods (F = 5.880) (Table 7). Freeze-drying significantly increased the water-holding capacity of the FDC and FHS pretreated samples compared to CD, HSCD, HSFD, HPCD, HPFD, HSHPD, and HSHPFD (*p* ≤ 0.05), while no significant differences were observed between freeze-drying and dehydration in the HS, HP, and HSHP pretreated samples at either drying method (Figure 2).

Carrot dietary fiber has a high water-holding capacity, ranging from 17.9 to 23.3 g water/g fiber compared to other vegetable fibers such as coconut, potato, and pea [8,21]. The water-holding capacity of fibers can be impacted by their tissue structure and structure shrinkage. Freeze-drying has been shown to affect the physicochemical and structural characteristics of foods. Removing water via sublimation during freeze-drying produces a highly porous structure and little shrinkage [14,15]. Freeze-dried spinach exhibits high porosity and increased surface area [37]. In contrast, carrot slices after conventional dehydration had a higher shrinkage rate (35.53%) than freeze-dried slices (20.83%) [38]. Hot air –drying has been shown to affect carrot tissue, causing the tissue to exhibit highly dense, less porous, and collapsed structures due to cellular damage from hot air–drying [39].

The ability of dietary fiber to hold water can be affected by its structure and how effectively it traps water. In the case of freeze-dried treatments, HS and HP pretreatments resulted in significant decreases in water-holding capacity (WHC) of 5.90% and 20.50%, respectively. The reduction in the WHC could be due to the change in insoluble and soluble fiber ratio and the reduction of insoluble to soluble fiber (Figure 2). Physically treated samples (HS, HP, and HSHP) showed a decrease in insoluble fiber but an increase in soluble fiber (Table 6). High-shear treatments could also be responsible for breaking up the fiber chains within carrot pomace. After milling, the WHC of grape pomace decreased from 2.52 g/g to 2.17 g/g [31]. However, an increase was seen in dehydrated HS and HP treatments. Thus, the length and degree of physical shearing can impact fiber degradation.

These results in this study support the findings that carrot pomace has a high water-holding capacity. Coconut pomace produced from coconut milk production is reported to have a high water-retention capacity of 5.4 g/g, which is lower than the WHC of carrot pomace in this study. The high WHC of carrot pomace suggests its potential uses in food applications as a functional ingredient.

#### 3.2.2. Fat-Binding Capacity

**Figure 3:**
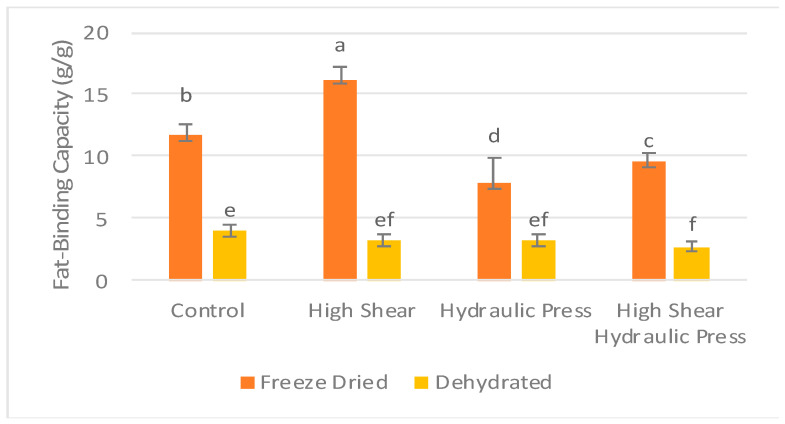
Impact of physical treatment methods on the fat-binding capacity of commercially produced carrot pomace. ^a–f^ Different letters within the same pretreatment indicate significant differences at *p* ≤ 0.05.

When looking at the effects of the drying method, pretreatment, or interaction, the drying method (F = 3058.060) had a more significant effect on FBC than the pretreatment (F = 170.72) and the interaction (F = 132.65) (Table 8). Freeze-drying significantly increased the fat-binding capacity (FBC) of carrot pomace compared to dehydration in all pretreatments (*p* < 0.05) (Figure 3). The FBC increased from 4.00 g/g in CD to 11.78 g/g in FDC, from 3.26 g/g in HSD to 16.6 g/g HSFD, from 2.88 g/g in HPD to 7.82 g/g in HSFD, and from 2.77 g/g in HSHPD to 9.59 g/g in HSHPFD.

Fat-binding capacity can be affected by various factors, such as plant polysaccharides, hydrophobic particle character, particle size, and the ratio of insoluble and soluble fibers [40]. The exposure of plant fibers to higher temperatures for an extended period can alter the physicochemical structure of polysaccharides and the hydrophobic nature of the particles [41]. Dehydration can result in the shrinking and deformation of fiber particles, causing a loss of the original shape, the formation of particle aggregates, and the reduction of space for water or fat to be absorbed [42]. During freeze-drying, intercellular water is frozen and removed as gas by sublimation. Therefore, freeze-dried particles can keep their original shape, showing larger sizes and more porous surfaces. Due to their larger volume and porosity, freeze-dried samples can bind more oil and water than conventionally dehydrated samples. The fat-binding capacity of dehydrated carrot pomace has been reported to be 3.95 ± 0.17 g/g [40], consistent with the values obtained from the present study. The fat-binding capacities of the fibrous residues of coconut fiber and banana powder are 5.30 g oil/g and 2.20 g oil/g, respectively [21]. Freeze-dried carrot pomace contained more total dietary fiber than conventionally dehydrated carrot pomace (Table 3). The composition of fibers plays a crucial role in the fat-binding capacity as well, as observed in date fiber concentrate, which showed a high oil-holding capacity (9.6–9.9 g oil/g). Date fiber concentrate was characterized by high levels of insoluble (81.3–84.7%) and soluble (6.7–7.69%) fibers [43]. Carrot pomace, a rich source of insoluble and soluble fibers, exhibits a higher fat-binding capacity, which could explain the higher FBC in carrot fibers than other fibers [40,44].

#### 3.2.3. Swelling Capacity (SC)

**Figure 4:**
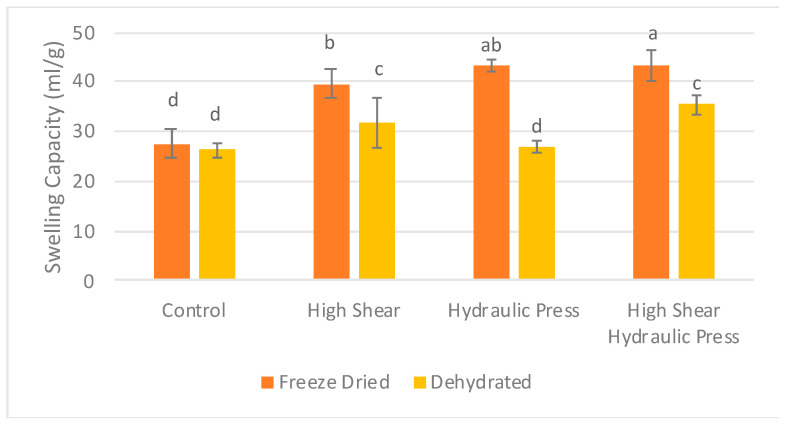
Impact of physical treatment methods on the swelling capacity of commercially produced carrot pomace. ^a–d^ Different letters within the same pretreatment indicate significant differences at *p* ≤ 0.05.

When looking at the effects of the drying method, pretreatment, and their interaction, the drying method (F = 384.312) had a more significant effect on SC than the pretreatment (F = 109.035) and interaction (F = 42.134) (Table 9). The results of the swelling capacity demonstrated that any physical treatment (HS, HP, and HSHP) could significantly increase the swelling capacity of both freeze-dried and dehydrated carrot pomaces (Figure 4). SC increased from 26.25 mL/g to 31.83 mL/g and 35.5 mL/g after HSD and HSHPD pretreatments, respectively, while SC increased from 27.50 mL/g to 43.33 mL/g after FDHP and FDHSHP pretreatments. These increases can be attributed to the enhanced exposure of hydrophilic groups in cellulose and hemicellulose, as well as the increased surface area and surface energy of particles after shearing [45].

The excessive grinding of vegetables may substantially disrupt the integrity of their dietary fiber chain, adversely affecting their hydration properties [46]. Asparagus pomace samples subjected to superfine grinding showed an initial increase in swelling capacity from 1.45 mL/g to 3.42 mL/g with a particle size reduction from 141.0 μm to 32.7 μm, which decreased thereafter as the particle size was further reduced to 6.1 μm [44]. Excessive micronation can lead to structural damage, reduce the total dietary fiber content, and consequently decrease the water-retention functionality of the fiber [45]. However, pretreatments showed benefits in increasing SC, possibly due to the breakage of long-chain dietary fibers into shorter-chain fibers and the porosity of fiber affecting its binding sites, resulting in enhanced hydration properties [47].

Soluble and insoluble fiber contents directly influence the swelling capacity and functionality of the product. The structural and chemical properties of fiber can play a role in the kinetics of water uptake. Water can be held by the capillary structures of dietary fiber due to surface tension. Additionally, water can interact with the molecular components of fiber through hydrogen bonds. The swelling capacities of orange and lemon fiber concentrates were 6.11 mL/g and 9.19 mL/g, respectively, corresponding to insoluble dietary fiber values of 54.0 g/100 g and 63.9 g/100 g, respectively [48]. The pectin in carrot pomace also has a greater water-holding capacity than cellulose fibers [40]. Pectin-rich citrus fibers have been shown to have a high water-swelling capacity [49]. The increased swelling capacity of pretreated carrot pomace could be due to the change in the insoluble to soluble fiber ratio after various pretreatments (Figure 4). Carrot pomace had a swelling capacity higher than that of coconut fibers [21]. The swelling capacity of carrot mash/peel has been reported to be 29.23 mL/g [17], comparable to both control pretreatments. The high swelling capacity of carrot pomace shows the benefit of dietary fibers with functional properties.

## 4. Conclusions

Freeze-drying was the most effective method compared to conventional dehydration in improving the functional properties of dried carrot pomace, including the water-holding, fat-binding, and swelling capacities. Freeze drying increased the water-holding capacity by 22% and the fat-binding capacity by 194%, with a greater retention of carotenoids. This is because of the lower temperatures and pressure during freeze-drying. Moreover, freeze-drying shows additional advantages by retaining up to 60% more carotenoid content within the carrot pomace. Physical pretreatments could also influence the functional properties of carrot pomace in combination with drying methods. Combining high-shearing and hydraulic pressing pretreatments increased the swelling capacity of freeze-dried carrot pomace and dehydrated carrot pomace compared to high-shearing or hydraulic pressing alone.

## Figures and Tables

**Table 1:** Pretreatment and drying methods applied to carrot pomace.

Samples *	Pretreatment	Drying Method
CD	No Pretreatment	Dehydration
CFD	No Pretreatment	Freeze-Drying
HSD	High-shear	Dehydration
HSFD	High-shear	Freeze-Drying
HPD	Hydraulic Press	Dehydration
HPFD	Hydraulic Press	Freeze-Drying
HSHPD	High-shear and Hydraulic Press	Dehydration
HSHPFD	High-shear and Hydraulic Press	Freeze-Drying

* CD = control/dehydration; CFD = control/freeze-drying; HSD = high-shear/dehydrated; HSFD = high-shear/freeze-drying; HPD = hydraulic press/dehydrated; HPFD = hydraulic press/freeze-drying; HSHPD = high-shear and hydraulic press/dehydrated; HSHPFD = high-shear and hydraulic press/freeze-drying.

**Table 2:** Impact of physical pretreatment methods on commercially produced carrot pomace’s moisture and solids content.

Pretreatments	Moisture Content (%)	Solids Content (%)
Control	94.45 ± 0.07 ^a^	5.55 ± 0.70 ^c^
HS	94.52 ± 0.38 ^a^	5.48 ± 0.38 ^c^
HP	85.68 ± 0.79 ^c^	14.31 ± 0.79 ^a^
HSHP	89.70 ± 0.28 ^b^	10.29 ± 0.28 ^b^

^a–c^ Different letters within columns indicate significant differences at *p* ≤ 0.05.

**Table 3:** Impact of physical treatment methods on the total carotenoid concentration of commercially produced carrot pomace.

Pretreatments	Freeze-Dried (μg/g)	Dehydrated (μg/g)
Control	100.07 ± 4.51 ^a^	44.91 ± 3.02 ^c^
HS	67.94 ± 3.80 ^b^	35.47 ± 1.35 ^d^
HP	90.11 ± 2.76 ^ab^	35.85 ± 1.19 ^d^
HSHP	67.79 ± 1.11 ^b^	48.72 ± 3.49 ^c^

^a–d^ Different letters within the same column indicate significant differences at *p* < 0.05.

**Table 4:** Significance of pretreatment and/or drying method on the fiber composition of dried carrot pomace.

	Source	Nparm	DF	Sum of Squares	F Ratio	Prob > F
TDF	Pretreatment	1	1	732.57	27.3662	<0.0001 *
	Drying Method	3	3	739.53	9.2088	0.0005 *
	Interaction	3	3	453.81	5.6510	0.0057 *
IDF	Pretreatment	1	1	175.12	33.1700	<0.0001 *
	Drying Method	3	3	976.41	61.6637	<0.0001 *
	Interaction	3	3	1258.89	79.5034	<0.0001 *
SDF	Pretreatment	1	1	182.07	9.9863	0.0049 *
	Drying Method	3	3	753.88	13.7828	<0.0001 *
	Interaction	3	3	308.19	5.6345	<0.0058 *

* Source with a significant impact (*p* < 0.05) on the fiber content (TDF, IDF, and SDF).

**Table 5:** Amylase neutral detergent fiber and acid detergent fiber composition of commercially produced dried carrot pomace.

Treatments	Freeze-Dried (g/100 g)	Dehydrated (g/100 g)
Amylase Neutral Detergent Fiber (aNDF)	37.57 ± 1.40 ^a^	23.13 ± 1.76 ^b^
Acid Detergent Fiber (ADF)	30.60 ± 0.30 ^a^	17.82 ± 1.32 ^b^

^a,b^ Different letters within the same row indicate significant differences at *p* ≤ 0.05.

**Table 6:** Impact of physical treatment methods on the fiber content of dehydrated and freeze-dried commercially produced carrot pomace (g/100 g).

Sample *	Total Dietary Fiber (TDF)	Insoluble Dietary Fiber (IDF)	Soluble Dietary Fiber (SDF)	Fiber Ratio (IDF:SDF)
CD	51.84 ± 7.08 ^c^	29.51 ± 2.66 ^c^	22.33 ± 5.30 ^cd^	2:1.5
CFD	71.87 ± 2.11 ^a^	55.38 ± 2.53 ^a^	16.49 ± 1.47 ^d^	3.4:1
HSD	54.68 ± 5.07 ^bc^	32.20 ± 0.79 ^d^	22.47 ± 4.40 ^bcd^	2:1.4
HSFD	65.27 ± 9.42 ^ab^	29.90 ± 2.12 ^c^	35.37 ± 4.30 ^a^	2:2.4
HPD	53.45 ± 2.58 ^bc^	28.95 ± 3.10 ^d^	24.49 ± 3.50 ^bc^	2:1.7
HPFD	56.21 ± 1.29 ^bc^	27.19 ± 1.01 ^d^	29.02 ± 0.48 ^ab^	2:1.9
HSHPD	67.17 ± 0.80 ^ab^	39.75 ± 2.10 ^bc^	27.42 ± 0.50 ^b^	2:1.4
HSHPFD	73.29 ± 5.78 ^a^	38.19 ± 2.28 ^b^	35.10 ± 3.59 ^a^	2:1.9

^a–d^ Different letters within the same column indicate significant differences at *p* ≤ 0.05. * CD = control/dehydration; CFD = control/freeze-drying; HSD = high-shear/dehydrated; HSFD = high-shear/freeze-drying; HPD = hydraulic press/dehydrated; HPFD = hydraulic press/freeze-drying; HSHPD = high-shear and hydraulic press/dehydrated; HSHPFD = high-shear and hydraulic press/freeze-drying.

**Table 7:** Significance of pretreatment and/or drying method on the water-holding capacity of dried carrot pomace.

Source	Nparm	DF	Sum of Squares	F Ratio	Prob > F
Pretreatment	1	1	504.284	43.215	<0.0001 *
Drying Method	3	3	5.734	1.474	0.2318
Interaction	3	3	68.612	5.880	0.0020 *

* Source with a significant impact (*p* < 0.05) on the water-holding capacity.

**Table 8:** Significance of pretreatment and/or drying method on the fat-binding capacity of dried carrot pomace.

Source	Nparm	DF	Sum of Squares	F Ratio	Prob > F
Pretreatment	1	1	133.360	170.725	<0.0001 *
Drying Method	3	3	796.255	3058.060	<0.0001 *
Interaction	3	3	103.637	132.675	<0.0001 *

* Source with a significant impact (*p* < 0.05) on the fat-binding capacity.

**Table 9:** Significance of pretreatment and/or drying method on the swelling capacity of dried carrot pomace.

Source	Nparm	DF	Sum of Squares	F Ratio	Prob > F
Pretreatment	1	1	950.307	109.035	<0.0001 *
Drying Method	3	3	1116.505	384.312	<0.0001 *
Interaction	3	3	367.224	42.134	<0.0001 *

* Source with a significant impact (*p* < 0.05) on the swelling capacity.

## Data Availability

The original contributions presented in the study are included in the article, further inquiries can be directed to the corresponding author.

## References

[1] FAO Global Initiative on Food Loss and Waste Reduction. https://www.fao.org/3/i7657e/i7657e.pdf.

[2] Buzby J. (2022). Food Waste and Its Links to Greenhouse Gases and Climate Change.

[3] Davis W., Lucier G. (2021). Vegetable and Pulses Outlook 2021.

[4] USDA-NASS QuickStats. https://quickstats.nass.usda.gov/.

[5] Yadav S., Pathera A.K., Islam R.U., Malik A.K., Sharma D.P. (2018). Effect of Wheat Bran and Dried Carrot Pomace Addition on Quality Characteristics of Chicken Sausage. Asian-Australas. J. Anim. Sci..

[6] Elik A., Yanık D.K., Göğüş F. (2020). Microwave-Assisted Extraction of Carotenoids from Carrot Juice Processing Waste Using Flaxseed Oil as a Solvent. LWT.

[7] Singh B., Panesar P., Nanda V. (2006). Utilization of Carrot Pomace for the Preparation of a Value Added Product. World J. Dairy Food Sci..

[8] Robertson J.A., Eastwood M.A., Yeoman M.M. (1979). An Investigation into the Physical Properties of Fiber Prepared from Several Carrot Varieties at Different Stages of Development. J. Sci. Food Agric..

[9] Thebaudin J.Y., Lefebvre A.C., Harrington M., Bourgeois C.M. (1997). Dietary Fibres: Nutritional and Technological Interest. Trends Food Sci. Technol..

[10] Nagai T., Reiji I., Hachiro I., Nobutaka S. (2003). Preparation and Antioxidant Properties of Water Extract of Propolis. Food Chem..

[11] Law C.L., Khan M.I.H., Wellard R.M., Mahiuddin M., Karim M.A. (2017). Cellular Level Water Distribution and Its Investigation Techniques. Intermittent and Nonstationary Drying Technologies: Principles and Applications.

[12] Berk Z. (2018). Food Process Engineering and Technology.

[13] Calín-Sánchez Á., Lipan L., Cano-Lamadrid M., Kharaghani A., Masztalerz K., Carbonell-Barrachina Á.A., Figiel A. (2020). Comparison of Traditional and Novel Drying Techniques and Its Effect on Quality of Fruits, Vegetables and Aromatic Herbs. Foods.

[14] Meda L., Ratti C. (2005). Rehydration of Freeze-Dried Strawberries at Varying Temperatures. J. Food Process Eng..

[15] Jia Y., Khalifa I., Hu L., Zhu W., Li J., Li K., Li C. (2019). Influence of Three Different Drying Techniques on Persimmon Chips’ Characteristics: A Comparison Study among Hot-Air, Combined Hot-Air-Microwave, and Vacuum-Freeze Drying Techniques. Food Bioprod. Process..

[16] Krokida M.K., Philippopoulos C. (2005). Rehydration of Dehydrated Foods. Dry. Technol..

[17] Amin S., Duval A., Jung S., Kang I. (2021). Valorization of Baby Carrot Processing Waste. J. Culin. Sci. Technol..

[18] AOAC (2012). Official Methods of Analysis of AOAC International.

[19] AACC (1991). Soluble, Insoluble, and Total Dietary Fiber in Foods and Food Products. AACC Approved Methods of Analysis.

[20] Total Dietary Fiber Assay Kit. https://www.megazyme.com/total-dietary-fiber-assay-kit.

[21] Raghavendra S.N., Rastogi N.K., Raghavarao K.S.M.S., Tharanathan R.N. (2004). Dietary Fiber from Coconut Residue: Effects of Different Treatments and Particle Size on the Hydration Properties. Eur. Food Res. Technol..

[22] Beuchat L.R. (1977). Functional and Electrophoretic Characteristics of Succinylated Peanut Flour Protein. J. Agric. Food Chem..

[23] Gopalan C., Ramasastry B.V., Balasubramanian S.C. (1991). Nutritive Valueof Indian Foods.

[24] Sharma S., Sagar N.A., Pareek S., Yahia E.M., Lobo M.G. (2018). Fruit and Vegetable Waste: Bioactive Compounds, Their Extraction, and Possible Utilization. Compr. Rev. Food Sci. Food Saf..

[25] Spotti M.J., Campanella O.H. (2017). Functional Modifications by Physical Treatments of Dietary Fibers Used in Food Formulations. Sens. Sci. Consum. Percept. Food Phys. Mater. Sci..

[26] Schultz A.K., Barrett D.M., Dungan S.R. (2014). Effect of Acidification on Carrot (*Daucus carota*) Juice Cloud Stability. J. Agric. Food Chem..

[27] Honda M., Takasu S., Nakagawa K., Tsuda T. (2021). Differences in Bioavailability and Tissue Accumulation Efficiency of (All-E)- and (Z)-Carotenoids: A Comparative Study. Food Chem..

[28] de la Rosa L., Alvarez-Parrilla E., Gonzalez-Agular G.A. (2010). Fruit and Vegetable Phytochemicals: Chemistry, Nutritional Value and Stability.

[29] Macura R., Michalczyk M., Fiutak G., Maciejaszek I. (2019). Effect of freeze-drying and air-drying on the content of carotenoids and anthocyanins in stored purple carrot. Acta Sci. Pol. Technol. Aliment..

[30] Gärtner C., Stahl W., Sies H. (1997). Lycopene Is More Bioavailable from Tomato Paste than from Fresh Tomatoes. Am. J. Clin. Nutr..

[31] Bender A.B.B., Speroni C.S., Moro K.I.B., Morisso F.D.P., Santos D.R., Silva L.P., Penna N.G. (2020). EfFfects of Micronization on Dietary Fiber Composition, Physicochemical Properties, Phenolic Compounds, and Antioxidant Capacity of Grape Pomace and Its Dietary Fiber Concentrate. LWT.

[32] Zhou X.L., Qian Y.F., Zhou Y.M., Zhang R. (2012). Effect of Enzymatic Extraction Treatment on Physicochemical Properties, Microstructure and Nutrient Composition of Tartary Buckwheat Bran: A New Source of Antioxidant Dietary Fiber. Adv. Mater. Res..

[33] Wang X., Xu Y., Liang D., Yan X., Shi H., Sun Y. (2015). Extrusion-Assisted Enzymatic Hydrolysis Extraction Process of Rice Bran Dietary Fiber. Proceedings of the 2015 ASABE Annual International Meeting.

[34] Bader Ul Ain H., Saeed F., Khan M.A., Niaz B., Rohi M., Nasir M.A., Tufail T., Anbreen F., Anjum F.M. (2019). Modification of Barley Dietary Fiber through Thermal Treatments. Food Sci. Nutr..

[35] de Moraes Crizel T., Jablonski A., de Oliveira Rios A., Rech R., Flôres S.H. (2013). Dietary Fiber from Orange Byproducts as a Potential Fat Replacer. LWT—Food Sci. Technol..

[36] Chau C.-F., Chen C.-H., Lee M.-H. (2004). Comparison of the Characteristics, Functional Properties, and in Vitro Hypoglycemic Effects of Various Carrot Insoluble Fiber-Rich Fractions. LWT—Food Sci. Technol..

[37] King V.A.E., Liu C.F., Liu Y.J. (2001). Chlorophyll Stability in Spinach Dehydrated by Freeze-Drying and Controlled Low-Temperature Vacuum Dehydration. Food Res. Int..

[38] Rajkumar G., Shanmugam S., Galvao M.D.S., Leite Neta M.T.S., Dutra Sandes R.D., Mujumdar A.S., Narain N. (2017). Comparative Evaluation of Physical Properties and Aroma Profile of Carrot Slices Subjected to Hot Air and Freeze Drying. Dry. Technol..

[39] Nahimana H., Zhang M. (2011). Shrinkage and Color Change during Microwave Vacuum Drying of Carrot. Dry. Technol..

[40] Sharoba A., Farrag M., Abd El-Salam A. (2013). Utilization of some fruits and vegetables wastes as a source of dietary fibers in cake making. J. Food Dairy Sci..

[41] Fernández-López J., Sendra-Nadal E., Navarro C., Sayas E., Viuda-Martos M., Alvarez J.A.P. (2009). Storage Stability of a High Dietary Fibre Powder from Orange By-products. Int. J. Food Sci. Technol..

[42] Fu W., Zhao G., Liu J. (2022). Effect of Preparation Methods on Physiochemical and Functional Properties of Yeast β-Glucan. LWT.

[43] Elleuch M., Besbes S., Roiseux O., Blecker C., Deroanne C., Drira N.E., Attia H. (2008). Date Flesh: Chemical Composition and Characteristics of the Dietary Fibre. Food Chem..

[44] Gao W., Chen F., Zhang L., Meng Q. (2020). Effects of Superfine Grinding on Asparagus Pomace. Part I: Changes on Physicochemical and Functional Properties. J. Food Sci..

[45] He S., Tang M., Sun H., Ye Y., Cao X., Wang J. (2019). Potential of Water Dropwort (*Oenanthe javanica* DC.) Powder as an Ingredient in Beverage: Functional, Thermal, Dissolution and Dispersion Properties after Superfine Grinding. Powder Technol..

[46] Zhao Y., Wu X., Wang Y., Jing R., Yue F. (2017). Comparing Physicochemical Properties of Hawthorn Superfine and Fine Powders. J. Food Process. Preserv..

[47] Meng X., Liu F., Xiao Y., Cao J., Wang M., Duan X. (2019). Alterations in Physicochemical and Functional Properties of Buckwheat Straw Insoluble Dietary Fiber by Alkaline Hydrogen Peroxide Treatment. Food Chem..

[48] Figuerola F., Hurtado M.L., Estevez A.M., Chiffelle I., Fernando A. (2005). Fibre Concentrates from Apple Pomace and Citrus Peel as Potential Fibre Sources for Food Enrichment. Food Chem..

[49] Huang J., Liao J., Qi J., Jiang W., Yang X. (2021). Structural and Physicochemical Properties of Pectin-Rich Dietary Fiber Prepared from Citrus Peel. Food Hydrocoll..

